# Differential effects of Cytomegalovirus carriage on the immune phenotype of middle-aged males and females

**DOI:** 10.1038/srep26892

**Published:** 2016-05-31

**Authors:** Marieke van der Heiden, Menno C. van Zelm, Sophinus J. W. Bartol, Lia G. H. de Rond, Guy A. M. Berbers, Annemieke M. H. Boots, Anne-Marie Buisman

**Affiliations:** 1 Centre for Infectious Disease Control (Cib), National Institute for Public Health and the Environment (RIVM), Bilthoven 3720 BA, The Netherlands; 2 Department of Rheumatology and Clinical Immunology, University of Groningen, University Medical Centre Groningen, Groningen 9700 RB, The Netherlands; 3 Department of Immunology, Erasmus MC, Rotterdam 3000 CA, The Netherlands; 4 Department of Immunology and Pathology, Central Clinical School, Monash University, Melbourne, Victoria 3004, Australia

## Abstract

The elderly population is more susceptible to infections as a result of an altered immune response, commonly referred to as immunosenescence. Cytomegalovirus (CMV)-infection associated changes in blood lymphocytes are known to impact this process, but the interaction with gender remains unclear. Therefore, we analysed the effects and interaction of gender and CMV on the absolute numbers of a comprehensive set of naive and memory T- and B-cell subsets in people between 50 and 65 years of age. Enumeration and characterisation of lymphocyte subsets by flow cytometry was performed on fresh whole blood samples from 255 middle-aged persons. CMV-IgG serostatus was determined by ELISA. Gender was a major factor affecting immune cell numbers. CMV infection was mainly associated with an expansion of late-differentiated T-cell subsets. CMV+ males carried lower numbers of total CD4+, CD4+ central memory (CM) and follicular helper T-cells than females and CMV− males. Moreover, CMV+ males had significantly lower numbers of regulatory T (Treg)-cells and memory B-cells than CMV+ females. We here demonstrate an interaction between the effects of CMV infection and gender on T- and B-cells in middle-aged individuals. These differential effects on adaptive immunity between males and females may have implications for vaccination strategies at middle-age.

Evidence is accumulating that the increased morbidity, risk for infections, and reduced vaccination responses in elderly are associated with changes in immune function^1^,^2^,^3^,^4^. Several heritable and non-heritable factors, such as chronological age, cytomegalovirus (CMV) infection, and gender have been documented to affect this process^5^, which is termed immunosenescence^1^,^2^,^3^,^4^.

Chronological age is primarily associated with alterations in the adaptive part of the immune system, especially the T-cell compartment. With age, thymic output of naive T-cells decreases to less than 10% of the original function by the age of 50 years^6^,^7^. This leads to increased peripheral replication of T-cells^7^,^8^, a reduction in naive T-cell numbers, and an expansion of memory T-cells^9^,^10^,^11^,^12^,^13^. Combined, these changes result in a diminished diversity of the T-cell receptor (TCR) repertoire, which may negatively impact on the recognition of novel antigens with age^14^. In addition, the numbers of several other lymphocytes are affected by age. Multiple studies have shown higher numbers of regulatory T-(Treg) cells^15^,^16^,^17^ and CD4+CD45RA+CD25^dim^ naive T-cells^8^,^18^ in elderly than in young adults. Moreover, an inverted CD4/CD8 T-cell ratio is observed with age, and has been proposed to be an immune risk indicator^19^,^20^. Finally, multiple studies showed an age-associated decline in the numbers of B-cells, both of the naive and the memory subsets^2^,^3^,^21^,^22^.

Multiple intrinsic and extrinsic factors may affect the immune status and infection with cytomegalovirus (CMV) has been associated with enhanced immunosenescence^23^,^24^,^25^. This herpes virus remains persistent upon primary infection and is actively suppressed by the immune system^23^. CMV infection primarily results in accumulation of late-differentiated memory T-cells, both in the CD4 and CD8 T-cell lineage^24^,^25^,^26^. These effects are already apparent in CMV-infected children^27^. CMV has limited effects on B-cell numbers, but might affect B-cell function as it is associated with high mutation frequencies in IgM and IgG transcripts^28^.

Gender is a major intrinsic factor that affects circulating immune cell numbers and immune function^17^,^19^,^29^,^30^. These effects can be mediated by hormone levels^30^,^31^,^32^,^33^, as well as by genes on sex chromosomes^33^. However, the impact of gender on naive and memory T- and B-cell numbers remains incompletely understood^29^. Recent studies suggest that T-cell senescence might be more pronounced in elderly men than in women^17^,^29^. Furthermore, the impact of persistent viruses, including CMV, might differ between males and females.

For a better understanding of immunosenescence, it is necessary to dissect the individual and combined effects of age, CMV infection and gender on numbers of circulating T- and B-cell subsets. Insights into these effects can be directly translated into early markers for immunosenescence. This knowledge is important in view of the general ageing of the population, because vaccines might be more effective when given before the onset of immunosenescence rather than at a specific age^34^,^35^.

In an effort to understand the effects and interaction of gender and CMV on the immune phenotype in a Dutch middle-aged population (defined as 50–65 years of age), we have enumerated a comprehensive set of T- and B-cell subsets including Treg cells, follicular helper T- (T_FH_) cells, and the ageing-associated CD4+CD45RA+CD25^dim^ naive T-cells. The characterisation of these ‘immune markers’ may help the identification of persons being at risk of impaired immune function and thereby higher susceptibility to disease. Our data reveal that CMV infection differentially affects the immune phenotype in middle-aged males and females.

## Results

### Characteristics of study participants

A total of 255 persons participated in the study with mean age: 57.7 (50–65) years, and of which 140 were male (54.9%). About half of the participants were seropositive for CMV. Baseline characteristics are shown in Table 1. Five participants were excluded from the analysis due to CMV status ambiguity. Mean age and BMI were similar between males and females, as well as between CMV+ and CMV− individuals.

### Effects of CMV on the absolute numbers of circulating leukocyte subsets

**Figure 1:**
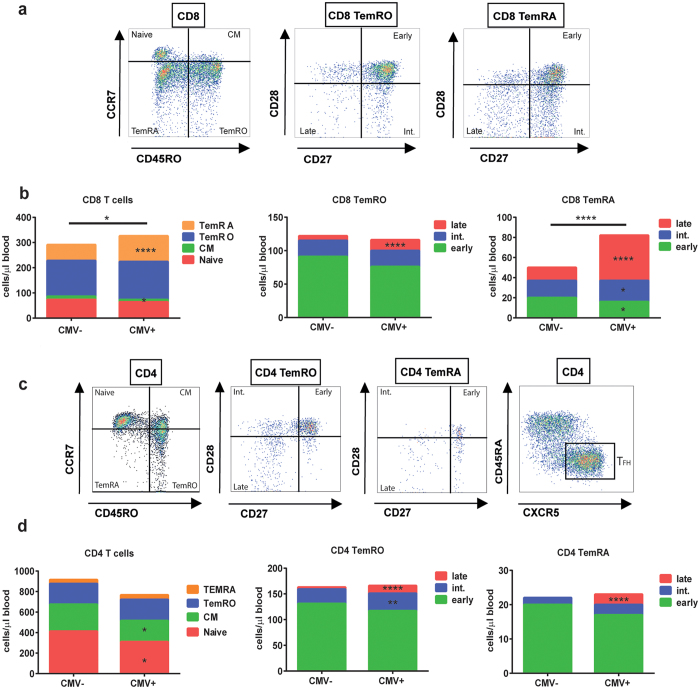
Effect of CMV on the CD8 and CD4 T-cell lineages. (**a)** Gating strategies for the CD8 T-cell subsets. A representative example is shown. (**b)** A cumulative schematic overview of the geometric mean values of absolute numbers of CD8 naive, CM, TemRO, and TemRA cells in CMV− and CMV+ participants. TemRO and TemRA cells are split into early, intermediate, and late differentiation subsets. (**c)** Gating strategies for the CD4 T-cell subsets. A representative example is shown. (**d)** A cumulative schematic overview of the geometric mean values of absolute numbers of CD4 naive, CM, TemRO, and TemRA cells in CMV− and CMV+ participants. TemRO and TemRA cells are were split into early, intermediate, and late differentiated subsets. The Mann Whitney U test was used for statistical analysis. *p < 0.05, **p < 0.01, ****p < 0.0001 (n = 250).

CMV infection significantly influenced 8 out of the 28 (28.5%) leukocyte subsets depicted in Table 1. A significantly lower number of NK-cells (p = 0.046) was observed in CMV+ persons (Table 1). In both the CD8 and CD4 T-cell lineage, the absolute numbers of late-differentiated effector memory (EM) TemRO and TemRA cells were significantly increased (all p < 0.0001) in CMV+ individuals (Fig. 1b,d). This resulted in significantly higher numbers of total CD8 T-cells (p = 0.036) in CMV+ individuals. The numbers of total CD4 T-cells were not different, because higher numbers of late-differentiated memory cells were accompanied by significantly lower numbers of naive (p = 0.023) and central memory (p = 0.030) CD4 T-cells (Table 1 and Fig. 1d). No differences were observed for T_FH_ and Treg cell numbers. Finally, as a consequence of the above-mentioned changes, the CD4/CD8 ratio was significantly lower in CMV+ individuals (p < 0.0001, Table 1).

### Effects of gender on the absolute numbers of circulating leukocyte subsets

Gender was associated with significant differences in 17 out of the 28 (60.7%) leukocyte subsets (Table 1). First, males showed significantly (p < 0.0001) lower numbers of total lymphocytes, resulting from low numbers in multiple subsets, especially the T-cells (p < 0.0001). As both numbers of CD4 T-cells (p < 0.0001) and CD8 T-cells (p = 0.006) were affected, no difference was observed in the CD4/CD8 ratios between males and females. In addition, males showed lower numbers of naive cells within both these lineages (both p < 0.0001). Except for the memory Treg cells, gender was associated with significant changes in the absolute numbers of all measured CD4 T-cell subsets, including the CD45RA+CD25^dim^ cells (p = 0.0008). Finally, males carried lower numbers of B-cells (p = 0.019) than females, which was mostly due to lower numbers of memory B-cells (CD27−, p = 0.007; CD27+, p = 0.004).

### Combined effects of CMV and gender on the absolute numbers of major lymphocytes subsets

To determine whether CMV infection was associated with differential effects on the immune phenotype between males and females, the participants were divided into four different groups: CMV− males, CMV+ males, CMV− females, and CMV+ females (Table 2). Mean age (57.7 years) and BMI (mean 25.9 range 18–39) were similar between the four study groups. Data on general health, lifestyle and biochemical parameters are reported in Supplementary Table 1. Furthermore, CMV infected males and females showed equal levels of CMV-specific IgG (Supplementary Fig.1).

Although CMV− males and females had similar total lymphocyte numbers in blood, these were significantly lower in CMV+ males than in CMV+ females (p < 0.0001; Table 2). This difference was mostly due to significantly lower numbers of CD4 T-cells in CMV+ males than in CMV+ females (p < 0.0001) and in CMV− males (p = 0.037) and to a lesser extent due to lower B-cell numbers compared to CMV+ females (p = 0.029). As a consequence, CMV+ males showed a significantly lower CD4/CD8 ratio than CMV− males (p < 0.0001) and CMV+ females (p = 0.011). Moreover, only within the CMV+ males, 5/61 participants had a CD4/CD8 ratio below 1, which has been referred to as an immune risk profile^19^,^20^. The lower numbers of B-cells in CMV+ males were mainly due to lower numbers of CD27− (p = 0.028) and CD27+ (p = 0.0066) memory B-cells (Table 2 and Supplementary Fig. 2).

### Combined effects of CMV and gender on the composition of the CD8 T-cell lineage

**Figure 2:**
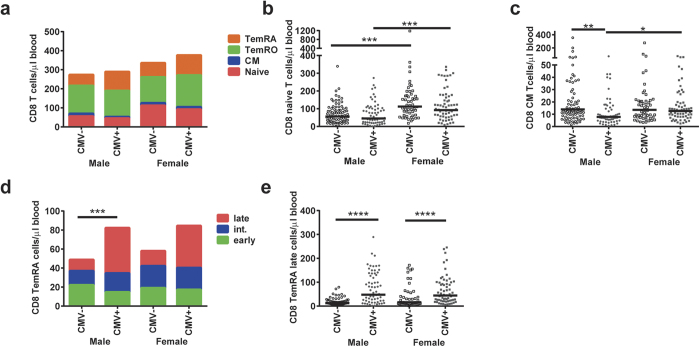
The combined effects of gender and CMV on the absolute numbers of CD8 T-cell subsets. (**a)** A cumulative schematic overview of the geometric mean values of absolute numbers of CD8 naive, CM, TemRO, and TemRA cells in CMV+ and CMV− males and females. **(b)** Absolute numbers of CD8 naive T-cells in CMV+ and CMV− males and females. (**c)** Absolute numbers of CD8 CM T-cells in CMV+ and CMV− males and females. (**d)** A cumulative schematic overview of the geometric mean values of absolute numbers of early, intermediate, and late differentiated subsets within the CD8 TemRA cells in CMV+ and CMV− males and females. The number of total CD8 TemRA cells was compared between the groups. (**e)** Absolute numbers of CD8 TemRA late cells in CMV+ and CMV− males and females. The Geometric mean is indicated in the graphs, and the Kruskal-Wallis test was used for statistical analysis. *p < 0.05, **p < 0.01, ***p < 0.001, ****p < 0.0001 after Bonferroni correction (n = 250).

Besides relatively stable absolute numbers of total CD8 T-cells, the composition of the CD8 T-cell lineage was affected by the combination of CMV and gender (Fig. 2a). Naive CD8 T-cell numbers were lower in males than in females, both in CMV+ (p = 0.0008) and CMV− (p = 0.0001) individuals (Fig. 2b). Moreover, the numbers of CD8 CM T-cells were significantly lower in CMV+ males than in CMV− males (p = 0.002) and in CMV+ females (p = 0.010) (Fig. 2c). Slightly different conclusions might be drawn when reporting on proportional data (Supplementary Fig. 3b). The higher numbers of late-differentiated CD8 TemRA cells in CMV infected individuals was equal in males and females (Fig. 2e). Furthermore, the distributions of CD8 TemRA early, intermediate, and late cells were similar between CMV-infected males and females (Fig. 2d).

### Combined effects of CMV and gender on the composition of the CD4 T-cell lineage

**Figure 3:**
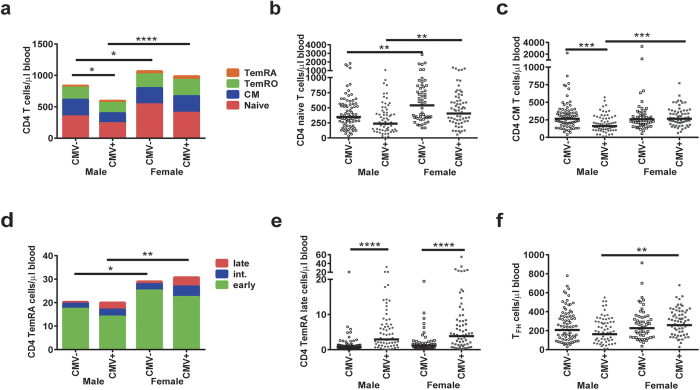
The combined effects of gender and CMV on the absolute numbers of CD4 T-cell subsets. (**a)** A cumulative schematic overview of the geometric mean values of absolute numbers of CD4 naive, CM, TemRO, and TemRA cells in CMV+ and CMV− males and females. The number of total CD4 T-cells was compared between the groups. (**b)** Absolute numbers of CD4 naive T-cells in CMV+ and CMV− males and females. (**c)** Absolute numbers of CD4 CM T-cells in CMV+ and CMV− males and females. (**d)** A cumulative schematic overview of the geometric mean values of absolute numbers of early, intermediate, and late differentiated subsets within the CD4 TemRA cells in CMV+ and CMV− males and females. The number of total CD4 TemRA cells was compared between the groups. (**e)** Absolute numbers of CD4 TemRA late cells in CMV+ and CMV− males and females. (**f)** Absolute numbers of T_FH_ cells in CMV+ and CMV− males and females. The Geometric mean is indicated in the graphs, and the Kruskal-Wallis test was used for statistical analysis. *p < 0.05, **p < 0.01, ***p < 0.001, ****p < 0.0001 after Bonferroni correction (n = 250).

CMV+ males carried lower numbers of CD4 T-cells, as a result of lower numbers of all CD4 subsets. Similar to naive CD8 T-cells, naive CD4 T-cell numbers were lower in males than in females, both in CMV+ (p = 0.005) and in CMV− (p = 0.007) donors (Fig. 3b). In addition, CD4 CM T-cells were lower in CMV+ males than in CMV− males (p = 0.0002) and in CMV+ females (p = 0.0002; Fig. 3c). However, different results were observed when considering proportional data on CD4 T-cell subsets due to the differences in total numbers of CD4 cells (Supplementary Fig. 3a). Numbers of total CD4 TemRA cells, however, were higher in females, independent of CMV infection. CMV+ males and females showed differences in the composition of CD4 TemRA cells, shifting from early to intermediate and late-stage cells within the CD4 TemRA compartment (Fig. 3d). The number of late-differentiated CD4 TemRA cells was equal in males and females (Fig. 3e). The overall finding of lower numbers of CD4 T-cell subsets in CMV+ males compared to CMV+ females was also visible for the T_FH_-cells (p = 0.005; Fig. 3f).

### Combined effect of CMV and gender on the absolute numbers of CD4+CD45RA+CD25^dim^ and Treg cells

**Figure 4:**
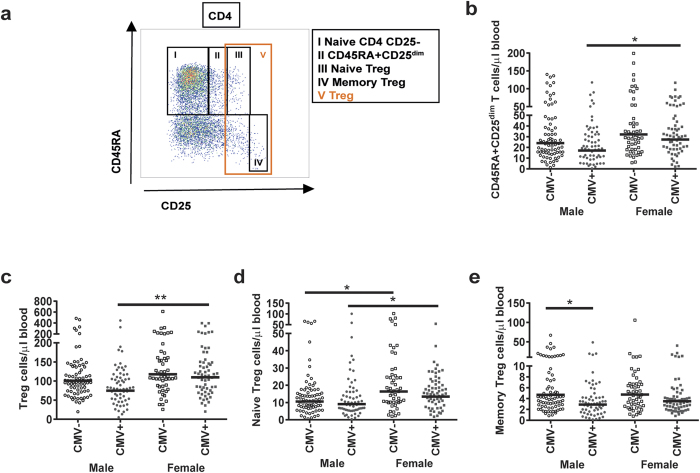
The combined effects of gender and CMV on the absolute numbers of CD4+CD45RA+CD25^dim^ T-cells and Treg cells. (**a)** Gating strategies for the different cell subsets. A representative sample is shown. (**b)** Absolute numbers of CD45RA+CD25^dim^ T-cells in CMV+ and CMV− males and females. (**c)** Absolute numbers of Treg cells in CMV+ and CMV− males and females. (**d)** Absolute numbers of naive Treg cells in CMV+ and CMV− males and females. (**e)** Absolute numbers of memory Treg cells in CMV+ and CMV− males and females. The Geometric mean is indicated in the graphs, and the Kruskal-Wallis test was used for statistical analysis, *p < 0.05, **p < 0.01, after Bonferroni correction (n = 250).

We next analysed the impact of CMV and gender on circulating total, naive and memory Treg subsets (Fig. 4a). Furthermore, we enumerated the peripherally expanded CD45RA+CD25^dim^ naive T-cell subset (population II; Fig. 4a) which accumulates with age^18^. In line with the lower numbers of other CD4 T-cells in CMV+ males, Treg cell (population V; Fig. 4a) numbers were also lower in CMV+ males than in CMV+ females (p = 0.004; Fig. 4c). Similar to CD4 and CD8 naive cells, naive Treg cells (population III; Fig. 4a) were lower in males than in females, both in CMV+ (p = 0.021) and in CMV− (p = 0.019) donors (Fig. 4d). No gender effect was seen for memory Tregs (population IV; Fig. 4a). Interestingly, CMV infection was only associated with significantly lower numbers of memory Treg cells in males (p = 0.038), and not in females (Fig. 4e). Numbers of CD4+CD45RA+CD25^dim^ cells were lower only in CMV+ males compared to CMV+ females (p = 0.016) (Fig. 4b).

### The interaction between gender and CMV status on the absolute numbers of lymphocytes

The results mentioned above on CD4 T-cells, CM T-cells, T_FH_, and Treg cells indicate a possible interaction between CMV infection and gender. To test this interaction a linear regression was performed that included the interaction CMV*Gender (Table 3 and Supplementary Table 2). This analysis confirmed the previously observed effects of either gender or CMV on lymphocyte numbers. Moreover, the numbers of CD4 T-cells were significantly affected by the combination of gender and CMV, with only CMV+ males showing lower CD4 T-cell numbers. The absolute numbers of both CD4 and CD8 CM, and T_FH_ cells were affected by CMV serostatus only in males. This analysis also revealed that in this cohort of middle-aged individuals, age affected the numbers of CD8 naive T-cells. Thus, in addition to CMV serostatus and age, the male gender is associated with phenotypic changes in lymphocytes, especially in combination with the persistence of CMV infection.

## Discussion

In this study, we show that both gender and CMV infection influence the immune phenotype of middle-aged persons (n = 255), an important population for possible future disease-preventing interventions. CMV infection was mainly associated with an accumulation of late-differentiated T-cells in both the CD4 and CD8 T-cell lineage. Gender was a major factor influencing the immune phenotype in middle-aged persons, with male participants demonstrating lower absolute numbers of lymphocytes within the majority of subsets. On top of the gender differences in naive T-cells, CMV+ males carried significantly lower numbers of CM CD4 and CD8 T-cells, as well as lower T_FH_, Treg and memory B-cells.

We show that middle-aged males have lower numbers of naive cells, both within the CD4 and CD8 T-cell lineage, which is in agreement with the previously reported accelerated drop in numbers of lymphocytes, especially naive cells, during ageing in males^17^. In addition, we found lower numbers of CD4+CD45RA+CD25^dim^ T-cells in the male participants. Recently, it was demonstrated that these CD4+CD45RA+CD25^dim^ T-cells accumulate with age in healthy individuals via peripheral expansion mechanisms and thereby represent a broad and functional reservoir of naive T-cells^8^,^18^. Several studies report a positive correlation between the number of naive T-cells and vaccine responses^36^,^37^, most likely due to a higher TCR diversity^12^,^14^. Since males show lower numbers of naive T-cells, this might point towards a lower capacity to respond to new antigenic challenges, a component of the immunosenescence process.

The association between CMV seropositivity and the accumulation of late-differentiated memory subsets in both the CD4 and CD8 T-cell lineage is in line with the current consensus in literature^24^,^26^,^27^,^29^. We demonstrate that the accumulation of these cells in CMV infected individuals was similar in males and females, which is in agreement with the study by *Di Benedetto et al.*^29^.

We found that CD4 T-cell numbers were reduced only in CMV+ males. Although this effect has been observed before in CMV+ individuals^26^, we are the first to demonstrate that this did not occur in females of middle-age. Consequently, CMV+ males show a lower CD4/CD8 ratio, which was even below 1 in some participants, indicative of an immune risk profile^19^. Importantly, when analysing proportional data, we and others^29^ found higher frequencies of CD8 T-cells within the T-cell compartment in CMV+ males (Supplementary Fig. 3). This has frequently been interpreted as an increase in CD8 T-cells rather than a decline in CD4 T-cells^29^. Here, we clearly show that CMV+ males have lower numbers of CD4 T-cells. Therefore, proportional data alone, without enumeration of cells, should be interpreted with caution.

The reduction in CD4 and CD8 CM T-cells in CMV+ males could be the result of less generation from their precursors, as these naive cells were reduced as well. Alternatively, it might indicate a higher turnover of CM T-cells towards more exhausted effector memory cell types in these males and thereby inducing a decrease in the capacity of developing memory immunity towards other pathogens. The latter explanation is in line with previous literature^38^ and the growing evidence of a higher susceptibility towards infections in older males^32^,^39^. However, our results are in contrast to results by *Di Benedetto et al.,* who did not find an effect of CMV on the relative proportions of CM cells in elderly^29^. This difference could be explained by the small sample size or the older age group as compared to our cohort.

In addition to lower numbers of naive and CM T-cells, CMV+ males also carried lower numbers of T_FH_ cells and memory B-cells. T_FH_ cells provide co-stimulation for activated B-cells in the germinal centre and thereby stimulate memory B-cell responses^40^. Moreover, T_FH_ cell numbers are associated with functional immunity and are reported to decrease with age^41^,^42^. Consequently, the parallel decrease in numbers of T_FH_ and memory B-cells is indicative for a reduced capacity of humoral immune responses in CMV+ males.

Although CMV+ males show multiple differences in immune cell numbers of which some might be indicative for accelerated immune ageing, males do not show higher numbers of (memory) Treg cells. This suggests that the pro- and anti-inflammatory balance in the immune system is still intact in these males. Previously, it has been shown in elderly that the balance between regulatory and effector CD4 T-cells is shifted towards a more suppressive, anti-inflammatory phenotype which is caused mainly by increases in memory Treg cells^43^. We have not observed this yet in the middle-aged group in our study. Furthermore, we found that the lower numbers of naive T-cells in males were accompanied by lower numbers of naive Treg cells. This aspect is in accordance with reports showing that naive Treg cells decrease with age^43^ and fits with the general decrease in naive cells with advancing age.

Despite the clear effects of gender and CMV infection on the immune phenotype in middle-aged persons, the variability in cell numbers between individuals was high. This variability indicates that most likely other heritable or non-heritable factors influence leukocyte numbers. Environmental factors, including infections with other persistent viruses, may have an impact on the blood lymphocyte numbers^27^. However, the current cohort was not found suitable for the analysis of many of these effects, because 92.6% of the participants were seropositive for EBV and the same applies for varicella zoster virus^44^,^45^. In addition, health status, BMI, smoking, and other lifestyle factors could have affected the numbers of cells in the various leukocyte subsets^46^,^47^,^48^,^49^. However, in our cohort, the general health status, smoking, and physical activity habits were similar between the four different groups (Supplementary Table 1). Therefore, it is unlikely that these factors have impacted on our results. Moreover, besides a high variation between individuals in BMI values, we did not observe any effects of BMI on the numbers of cells in the lymphocyte subsets (data not shown). This could be related to the specific age group that we studied. Still, it should be noted that the self-reported weight and height introduced additional variation and that our study might not be fully powered to conclude on effects of BMI.

The effects of CMV infection in females were limited to the expansion of late-differentiated cell subsets, whereas CMV-infected males also showed lower numbers in CD4 T-cell subsets, as well as CD8 CM T-cells. These findings suggest that the female immune system might be better able to control CMV at middle-age. This effect is most likely mediated by differential effects of sex hormones on the immune response, since estradiol is found to mainly enhance immune responses, whereas testosterone mainly has a suppressive effect^30^,^32^. In this cohort, dehydroepiandrosterone sulphate (DHEAs) levels, the precursor for most major sex hormones^50^, were higher in males than in females (Supplementary Table 1). For both genders DHEAs levels showed a slight decrease between the age of 50 and 65 (data not shown). These findings are in agreement with existing knowledge^51^,^52^. Moreover, it is most likely that not all females in this cohort have been through the menopause, which might influence their estrogen levels^33^. Still, this will not have affected our results much, because the age distribution and DHEAs levels were similar between the CMV+ females and CMV− females. It is possible that CMV affects the immune phenotype more in elderly females, when the protective effect of estrogen subsides. The more extensive effect of CMV on the immune phenotype in middle-aged males might be mediated by high testosterone levels that suppress their immune response towards CMV reactivation. This outcome would be in line with the growing evidence of a higher susceptibility towards infections in males and the higher resistance to infectious diseases in females^32^. The latter could probably be related to better management of infection and preserved capacity to recognize novel pathogens in females of middle-age.

Our data reveal early signs of immunosenescence in middle aged, CMV+ males. Based on the accelerated immune ageing in males^17^ and an enlarged effect of CMV at elderly age^26^ as reported by others, we expect a progressive increase of immunosenescent features with ageing in CMV+ males. Differences between CMV+ and CMV− females may become more apparent beyond the age of 65. Follow-up studies are required to assess if these gender differences in more elderly study groups will remain to exist or even progress.

In conclusion, we found a direct association between gender, CMV and the immune phenotype of middle-aged individuals. The reductions in circulating CCR7+ naive and central memory T-cells as well as memory B-cells could have a direct impact on adaptive immune responses and are suggestive of early immunosenescence in CMV+ males, a notion that would merit further investigation. Since middle-aged individuals are an interesting population for future vaccine interventions, our findings are of direct relevance for future strategies regarding vaccination of the ageing population.

## Methods

### Study subjects and blood sampling

Peripheral blood was collected after written informed consent was obtained from 255 healthy middle-aged persons, aged 50–65 years of age, who were equally distributed over three 5-year intervals: 50–54, 55–59, and 60–65 years. The study was approved by the Medical Ethical Committee: Verenigde Commissie Mensgeboden Onderzoek (VCMO) in Nieuwegein, the Netherlands and registered at the Dutch trial register (NTR4636). All procedures were in accordance with the Declaration of Helsinki.

Subjects were excluded if they had fever or used antibiotics within the last 14 days, had a serious immune related disease such as cancer, received immunosuppressive treatment within the last 3 months (e.g. steroids), had a known or suspected immune deficiency, a coagulation disorder or a neurologic disorder, used hormone treatment, or were administered blood products within the last 6 months. All participants completed a short questionnaire concerning health status, medication use, smoking, and physical activity. Weight and height obtained from the short health questionnaire were used to calculate the Body Mass Index (BMI) according to the formula BMI = Weight (kg)/Height (m)^2^.

Blood samples were collected during evening hours in tubes containing lithium heparin (BD Biosciences, Franklin Lakes, New Jersey) for detailed cellular immune phenotyping within 24 hours after collection. Additionally, serum was collected using serum clotting tubes (BD Biosciences) and processed within 6 hours for storage at −20 °C.

### CMV and EBV serology

Serum CMV IgG was determined by an enzyme-linked immunoassay (ETI-CYTOK-G Plus, P002033, Diasorin, Salugga, Italy) according to the manufacturer’s indications. The threshold for CMV seropositivity was 0.4 IU/ml. Serum Epstein Barr Virus (EBV)-capsid antigen IgG was determined by an enzyme-linked immunoassay (EUROIMMUN, Lubeck, Germany) according to the manufacturer’s indications. The threshold for EBV seropositivity was 20 relative units (RU)/ml.

### Biochemical parameter measurements

Serum levels of C-reactive protein (CRP), Rheumatoid Factor (RF), and Reactive Oxygen Metabolites (ROM) were measured with a clinical auto-analyser (Dx5, Beckman-Coulter). The kits used were from Beckman-Coulter, Fullerton, CA (CRP), Roche Diagnostics, Almere, The Netherlands (RF), and Diacron, Grosseto, Italy (ROM). Dehydroepiandrosterone Sulphate (DHEAs), a precursor for most major sex hormones^53^, was measured using the kit and immuno-analyser Access-2 from Beckman Coulter.

### Flow cytometric immune phenotyping

The absolute numbers of lymphocytes, CD3+ T-cells, B-cell subsets, NK-cells, monocytes, and granulocytes were determined with a lyse-no-wash protocol using TruCOUNT tubes (BD Biosciences, San Jose, CA, USA). The following fluorochrome-conjugated antibodies were used: CD3(UCHT1)-BV711, CD16(B73.1)-PE, and CD38(HB7)-APC-H7 (all from BD Biosciences), CD45(GA90)-OC515 and CD56(C5.9)-PE (both from Cytognos, Salamanca, Spain), CD27(M-T271)-BV421 and IgD(IA6-2)-FITC (both from Biolegend, San Diego, CA), and CD19(J3-119)-PE-Cy7 (Beckman Coulter, Fullerton, CA).

Detailed immune phenotyping of T-cell subsets was performed separately in fresh whole blood samples using additional antibodies: CD4(RPA-T4)-BV510, CD45RA(HI100)-BV605 and CD28(CD28.2)-PerCP- Cy5.5 (all from Biolegend), CCR7(150503)-PE-CF594, CD8(SK1)-APC-H7, CD25(2A3)-FITC, and TCRgd(11F2)-PE-Cy7 (all from BD Biosciences), and CXCR5(51505)-APC (R&D systems, Minneapolis, MN). Absolute numbers of T-cell subsets were calculated using the CD3+ T-cell numbers from the TruCOUNT analysis. Gating strategies for T-cells^54^, Treg cells^55^, and B-cells^56^ were applied as described previously, and shown in Figs 1a,c and 4a and Supplementary Fig. 2A, respectively. In short, CCR7+ T cells were separated into CD45RA+ naive and CD45RA- central memory (CM) subsets as described by *Sallusto et al.*^57^. Furthermore, CCR7- effector memory T cells (Tem) were separated into CD45RA- TemRO and CD45RA+ TemRA cells. Within TemRO and TemRA, early, intermediate and late subsets were defined on the basis of differential expression of CD27 and CD28, as described by *Appay et al.*^58^. In previous studies, CD45RA- T cells were confirmed to be CD45RO+^27^. Flow cytometric analyses were performed on a 4-laser LSRFortessa (BD Biosciences) using standardized measurement settings as described by *Kalina T et al.*^59^, and data analysis using FacsDiva V8 (BD Biosciences) and FlowJo V10 (FlowJo company, Ashland, OR).

### Statistics

Normal distribution of the data was tested with the D’Agostino & Pearson omnibus normality test.

The Mann Whitney U test was used to compare the CMV+ and CMV− groups, and males versus females on single immune cell subsets. To determine significant differences of one cell subset between two groups, p-values < 0.05 were considered significant. CMV+ males, CMV− males, CMV+ females, and CMV− females were compared for every immune cell subset using the Kruskal-Wallis test adjusted for multiple comparisons with the Bonferroni correction. The following comparisons were made: CMV+ males vs CMV+ females, CMV+ males vs CMV− males, CMV+ females vs CMV− females, CMV− females vs CMV− males. These statistical tests were performed in GraphPad Prism v6.05 for Windows (GraphPad Software Inc., La Jolla, CA). To conclude whether gender, CMV or the interaction between gender and CMV had an effect on the immune phenotype, a multiple comparison correction was included, since 36 immune cell subsets were tested. Only p-values < 0.0014 (p = 0.05/36) were considered significant. The statistical tests were supplemented with an Enter linear regression method in SPSS V22.0 to determine the individual effects of CMV status, gender, age, and the interaction between CMV status and gender on the immune cell subsets. Non-normally distributed data were log-transformed. To confirm the differential effects of CMV in males and females, an interaction term, gender*CMV, was included in the analysis. In this model, the p-value indicates whether a variable was significantly associated with the absolute number of the respective immune cells. The β coefficient indicates the strength of the association; the higher the value of β, the larger the deviation between groups that were compared. A negative value indicates a lower number of cells within males, CMV+ individuals, or CMV+ males, whereas a positive value indicates a higher absolute number of cells in these groups. The R^2^ of the model explains the strength of the model in predicting the absolute number of the respective immune cells; the closer the R^2^ is to 1, the stronger the predictive value of the model.

## Additional Information

**How to cite this article**: van der Heiden, M. *et al.* Differential effects of Cytomegalovirus carriage on the immune phenotype of middle-aged males and females. *Sci. Rep.* **6**, 26892; doi: 10.1038/srep26892 (2016).

## Supplementary Material

Supplementary Information

## Figures and Tables

**Table 1:** Individual effects of CMV and gender on the absolute numbers of different leukocyte subsets.

	CMV−	CMV+	Male	Female
**Demographic factors**				
No. (%)	127 (50.8)	123 (49.2)	140 (54.9)	115 (45.1)
Mean age in years (range)	57.6 (50–65)	57.8 (50–65)	57.8 (50–65)	57.5 (50–65)
Mean BMI (range)	25.7 (19–39)	26.0 (18–36)	25.8 (19–35)	25.9 (18–39)
**Leukocyte subsets (cells/μl blood)**				
Granulocytes	2275 [2075–2495]	2198 [2018–2394]	2249 [2060–2457]	2240 [2041–2459]
Monocytes	198.5 [182.6–215.8]	182.5 [168.8–197.3]	192.8 [178.0–208.8]	188.5 [173.0–205.5]
NK cells	143.5 [126.0–163.3]	118.2 [103.0–135.6]*	131.6 [115.7–149.8]	131.3 [114.3–150.9]
Lymphocytes	2179 [2039–2329]	2174 [2030–2328]	1937 [1799–2087]	2367 [2204–2541]****
T cells	1548 [1428–1678]	1428 [1293–1577]	1317 [1201–1445]	1732 [1599–1875]****
γδ T cells	18.7 [16.01–21.85]	17.4 [14.95–20.17]	16.2 [14.0–18.8]	20.6 [17.6–24.1]*
CD4/CD8 ratio	3.3 [3.0–3.6]	2.5 [2.3–2.7]****	2.7 [2.5–2.9]	3.1 [2.8–3.3]
CD8 T cells	331.7 [299.2–367.8]	368.0 [328.4–412.3]*	317.8 [285.0–354.3]	393.8 [354.9–437.0]*
CD8 naive	74.0 [63.3–86.4]	64.7 [53.4–78.4]	51.3 [43.2–61.0]	100.9 [87.2–116.8]****
CD8 CM	13.8 [11.7–16.2]	10.0 [8.5–11.7]*	11.1 [9.3–13.1]	13.0 [11.2–15.1]
CD8 TemRO	142.4 [126.8–160.0]	151.3 [134.7–169.9]	144.6 [128.7–162.5]	151.9 [135.5–170.2]
CD8 TemRA	63.2 [54.9–72.9]	102.3 [87.8–119.2]****	72.3 [62.5–83.7]	88.9 [75.9–104.0]
CD4 T cells	1084 [994.5–1181]	911.3 [817.0–1016]	852.0 [769.4–943.3]	1205 [1109–1309]****
CD4 naive	414.0 [363.6–471.4]	310.3 [265.5–362.7]*	290.6 [251.1–336.3]	460.2 [405.3–522.5]****
CD4 CM	262.1 [234.8–292.6]	206.7 [182.9–233.5]*	213.8 [188.4–242.7]	260.5 [235.3–288.4]*
CD4 TemRO	202.2 [184.0–222.2]	206.3 [182.7–232.8]	181.2 [162.9–201.5]	239.5 [216.4–265.1]***
CD4 TemRA	38.1 [32.8–44.3]	44.5 [37.8–52.3]	32.2 [28.0–37.0]	53.9 [45.9–63.3]****
T_FH_ cells	214.3 [189.4–242.5]	205.3 [178.9–235.6]	186.4 [162.7–213.5]	241.7 [215.9–270.6]*
Treg cells	107.3 [97.5–118.1]	90.7 [80.4–102.4]	89.0 [79.7–99.4]	113.6 [102.4–126.0]***
naive Treg	12.6 [10.7–14.7]	11.08 [9.5–13.0]	9.9 [8.4–11.5]	14.7 [12.7–17.0]***
memory Treg	4.7 [4.0–5.6]	3.2 [2.7–3.8]**	3.9 [3.2–4.7]	4.0 [3.4–4.7]
CD4+CD45RA+CD25^dim^	27.0 [23.1–31.5]	21.7 [18.3–25.6]	20.3 [17.3–23.9]	29.7 [25.5–34.4]***
B cells	286.7 [257.8–318.9]	265.8 [241.0–293.0]	259.2 [234.4–286.5]	301.8 [271.8–335.0]*
transitional	9.5 [8.1–11.2]	7.7 [6.5–9.1]	7.9 [6.8–9.1]	9.7 [8.1–11.5]
naive mature	175.3 [154.2–199.2]	162.2 [142.1–185.1]	160.9 [144.2–179.4]	183.2 [161.6–207.7]
natural effector	22.1 [18.4–26.6]	26.6 [22.3–31.8]	21.7 [18.7–25.2]	23.8 [20.0–28.5]
CD27− memory	15.5 [13.1–18.3]	15.3 [13.4–17.6]	14.8 [12.8–17.0]	19.2 [16.8–22.0]**
CD27+ memory	32.8 [28.1–38.3]	36.6 [32.1–41.8]	29.0 [25.5–33.0]	36.7 [32.0–42.1]**

Geometric mean of cells/μl blood [95% CI]. CMV− and CMV+ persons were compared, as well as males and females. *p < 0.05, **p < 0.01, ***p < 0.001, ****p < 0.0001. Bold numbers indicate significant differences within one subset. After a multiple testing correction only values with p < 0.0014 are considered significant. Significant differences after correction for multiple testing are underlined.

**Table 2:** Combined effects of CMV and gender on the absolute numbers of different leukocyte subsets.

	Male CMV−	Male CMV+	Female CMV−	Female CMV+
**Demographic factors**				
No. (%)	76 (30)	61 (24)	51 (20)	62 (25)
Mean age (range)	57.9 (50–65)	57.9 (50–65)	57.2 (50–65)	57.8 (50–65)
Mean BMI (range)	25.8 (19–39)	26.1 (18–36)	25.7 (19–35)	25.9 (19–35)
**Leukocyte subsets (cells/μl blood)**				
Lymphocytes	2043 [1854–2251]	1820 [1624–2041]^****a^	2367 [2078–2696]	2405 [2223–2602]
NK cells	145.9 [122.8–173.5]	110.1 [90.7–133.6]	139.8 [114.2–171.2]	126.7 [103.9–154.5]
Monocytes	202.1 [182.0–224.4]	177.8 [158.6–199.3]	193.0 [167.5–222.5]	187.2 [167.9–208.8]
Granulocytes	2392 [2140–2675]	2059 [1810–2342]	2105 [1792–2473]	2344 [2092–2625]
T cells	1422 [1292–1567]^*b^	1164 [991.8–1367]^**** a^	1756 [1532–2012]	1746 [1583–1927]
CD4 T-cells	989.4 [891.0–1099]*b	701.1 [589.4–833.8]^****a/*c^	1242 [1076–1432]	1180 [1065–1306]
CD8 T-cells	304.7 [267.3–347.3]	329.0 [274.8–394.0]	376.5 [318.8–444.7]	410.9 [357.4–472.3]
CD4/CD8 ratio	3.2 [2.9–3.6]	2.1 [1.9–2.4]^*a/****c^	3.3 [2.9–3.8]	2.9 [2.5–3.2]
Treg cells	100.8 [89.5–113.6]	74.7 [62.0–90.0]**a	117.8 [100.2–138.5]	109.8 [95.2–126.8]
γδ T-cells	16.3 [13.4–19.8]	16.3 [13.0–20.4]	22.9 [17.8–29.5]	18.5 [15.1–22.7]
B cells	273.9 [237.3–316.1]	235.9 [207.2–268.6]^*a^	306.9 [261.2–360.6]	298.8 [259.0–344.7]
transitional	8.2 [6.6–10.1]	7.1 [5.7–8.8]	12.0 [9.3–15.3]	8.4 [6.5–10.7]
naive mature	167.8 [143.4–196.4]	149.1 [129.1–172.3]	194.3 [162.5–232.3]	174.0 [145.4–208.1]
natural effector	21.6 [18.0–26.0]	20.0 [15.6–25.8]	20.9 [15.7–27.9]	27.3 [21.9–34.1]
CD27− memory	15.7 [12.6–19.5]	13.3 [11.3–15.6]^*a^	19.7 [15.8–24.5]	19.2 [16.2–22.7]
CD27+ memory	29.7 [24.8–35.5]	27.4 [22.9–32.9]^**a^	33.3 [26.2–42.2]	41.5 [35.6–48.3]

Geometic mean of cells/μl blood [95% CI]. *p < 0.05, **p < 0.01, ***p < 0.001, ****p < 0.0001; a. Male CMV+ versus Female CMV+; b. Male CMV− versus Female CMV−; c. Male CMV+ versus Male CMV−; Bold numbers indicate significant differences within one subset. After a multiple testing correction only values with p < 0.0014 are considered significant. Significant differences after correction for multiple testing are underlined.

**Table 3:** Linear regression analysis for the absolute numbers of different immune cell subsets.

Leukocyte subsets	Predicting variable	p-value	β coefficient	R^2^ model
Lymphocytes	CMV status	0.869	0.015	0.072
	**Gender**	0.046*	**−0.176**	
	Gender*CMV	0.227	−0.129	
	Age	0.375	0.055	
T cell	CMV status	0.928	−0.008	0.108
	**Gender**	0.016*	**−0.209**	
	Gender*CMV	0.124	−0.162	
	Age	0.422	0.049	
CD4 T cell	CMV status	0.580	−0.049	0.153
	**Gender**	0.015*	**−0.206**	
	Gender*CMV	0.029*	**−0.223**	
	Age	0.452	0.044	
CD4 naive	**CMV status**	0.048*	**−0.178**	0.121
	**Gender**	0.002*	**−0.272**	
	Gender*CMV	0.652	−0.047	
	Age	0.547	0.036	
CD4 CM T cell	CMV status	0.820	0.021	0.102
	Gender	0.801	0.022	
	Gender*CMV	0.001*	**−0.342**	
	Age	0.855	0.011	
CD4 TemRO	CMV status	0.135	0.138	0.074
	Gender	0.237	−0.104	
	Gender*CMV	0.032*	**−0.230**	
	Age	0.801	0.016	
CD4 TemRO late	**CMV status**	0.000*	**0.585**	0.305
	Gender	0.612	0.039	
	Gender*CMV	0.586	−0.050	
	Age	0.732	−0.018	
CD4 TemRA	CMV status	0.444	0.070	0.083
	**Gender**	0.003*	**−0.263**	
	Gender*CMV	0.858	−0.019	
	Age	0.555	0.036	
CD4 TemRA late	**CMV status**	0.000*	**0.430**	0.260
	**Gender**	0.044*	**−0.161**	
	Gender*CMV	0.403	0.080	
	Age	0.485	0.039	
T_FH_	CMV status	0.342	0.089	0.053
	Gender	0.479	−0.063	
	Gender*CMV	0.045*	**−0.217**	
	Age	0.518	0.040	
CD8 T-cell	CMV status	0.440	0.072	0.038
	Gender	0.057	−0.171	
	Gender*CMV	0.940	−0.008	
	Age	0.766	−0.019	
CD8 naive	CMV status	0.310	−0.089	0.169
	**Gender**	0.000*	**−0.340**	
	Gender*CMV	0.870	−0.017	
	**Age**	0.001*	**−0.196**	
CD8 CM T cell	CMV status	0.656	−0.041	0.070
	Gender	0.908	0.010	
	Gender*CMV	0.029*	**−0.235**	
	Age	0.254	0.071	
CD8 TemRO	CMV status	0.110	0.152	0.015
	Gender	0.545	0.055	
	Gender*CMV	0.114	−0.175	
	Age	0.712	0.023	
CD8 TemRO late	**CMV status**	0.000*	**0.585**	0.305
	Gender	0.612	0.039	
	Gender*CMV	0.586	−0.050	
	Age	0.732	−0.018	
CD8 TemRA	**CMV status**	0.025*	**0.206**	0.089
	Gender	0.097	−0.145	
	Gender*CMV	0.350	0.099	
	Age	0.622	0.030	
CD8 TemRA late	**CMV status**	0.000*	**0.412**	0.259
	Gender	0.132	−0.119	
	Gender*CMV	0.183	0.128	
	Age	0.388	0.048	

Linear (Enter) regression was performed on log-transformed data by using the variables CMV status (0 = seronegative, 1 = seropositive), Gender (0 = female, 1 = male), Gender* CMV status, and Age (years). Bold numbers indicate significant differences within one subset. *p < 0.05. After a multiple testing correction only values with p < 0.0014 are considered significant, which are underlined. The p-value indicates the association with the absolute number of immune cells, the β coefficient indicates the strength of the association, and the R2 explains the strength of the model in predicting the absolute number of immune cells.
