# Supplementary material for: Differential effects of Cytomegalovirus carriage on the immune phenotype of middle-aged males and females
Source: Sci Rep. 2016 May 31;6:26892. doi: 10.1038/srep26892 (PMC4886678; doi:10.1038/srep26892)
Supplement: Supplementary Information [file srep26892-s1.pdf]

Supplementary information

Differential effects of Cytomegalovirus carriage on the immune phenotype of middle-aged males and females.

Marieke van der Heiden[1,2], Menno C. van Zelm[3,4], Sophinus J.W. Bartol[3], Lia G.H. de Rond[1], Guy A.M. Berbers[1], Annemieke M.H. Boots[2], Anne-Marie Buisman[1]

Supplementary Figures

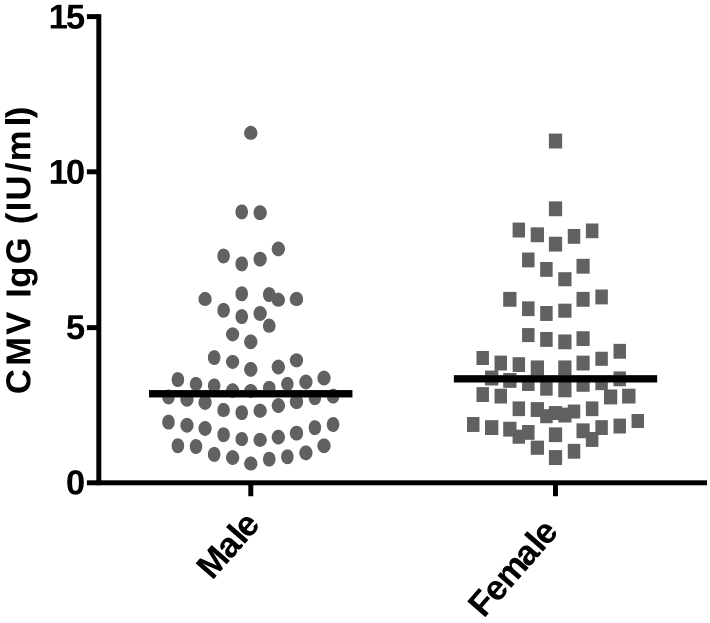

Supplementary Figure 1. The CMV IgG titers in males and females
The geometric mean is shown. The Mann Whitney U test was used for statistical analysis (n=123).

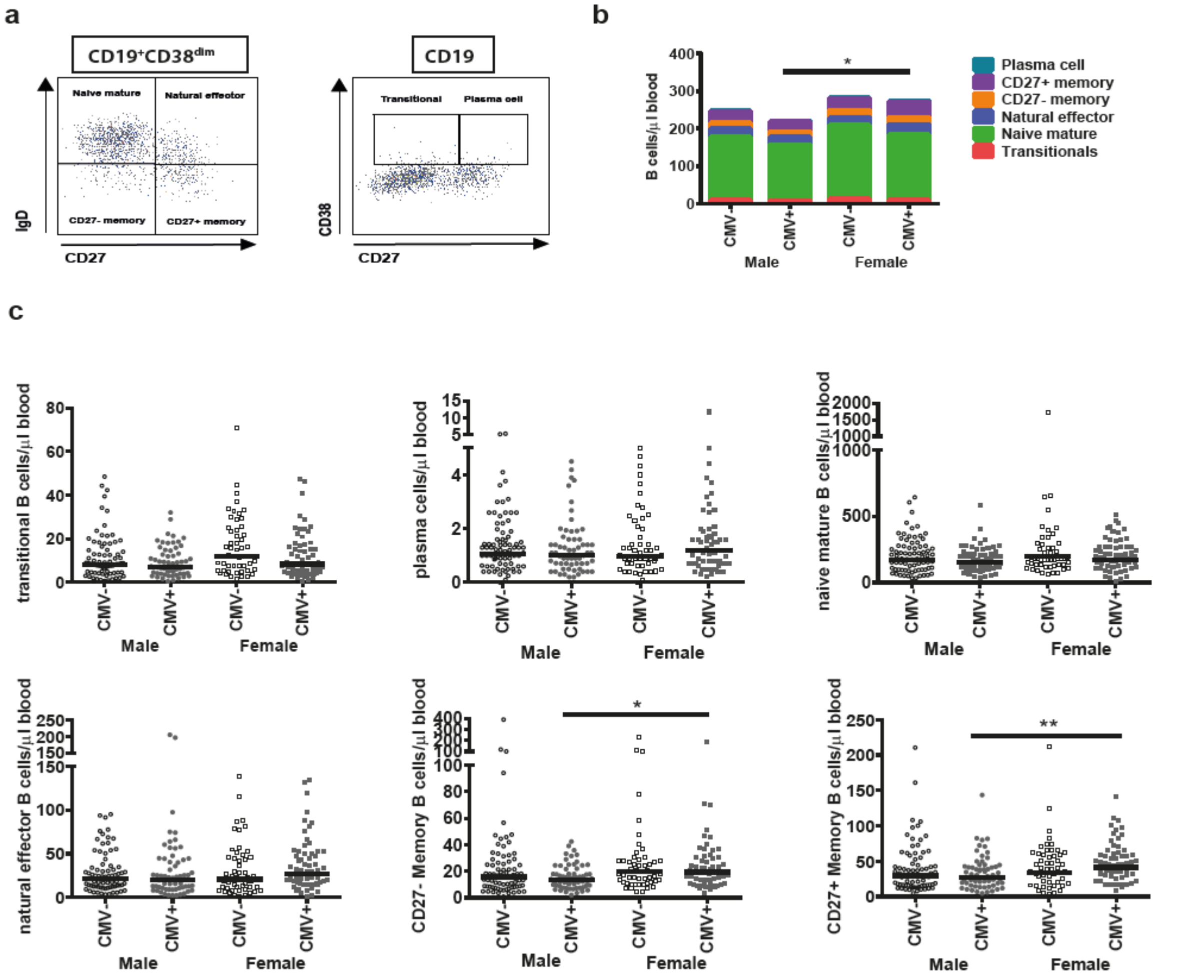

Supplementary figure 2. The combined effects of gender and CMV on the absolute numbers of B cell subsets

a) Gating strategy of the different B cell subsets. A representative sample is shown. **b)** A cumulative schematic overview of the geometric mean values of absolute numbers of different B cell subsets. **c)** Absolute numbers of transitional, plasma, naive mature, natural effector, CD27- memory, and CD27+ memory B cells in the four groups. The Geometric mean is indicated in the graphs, and the Kruskal - Wallis test was used for statistical analysis, * p<0.05, ** p<0.01 after Bonferroni correction (n=250).

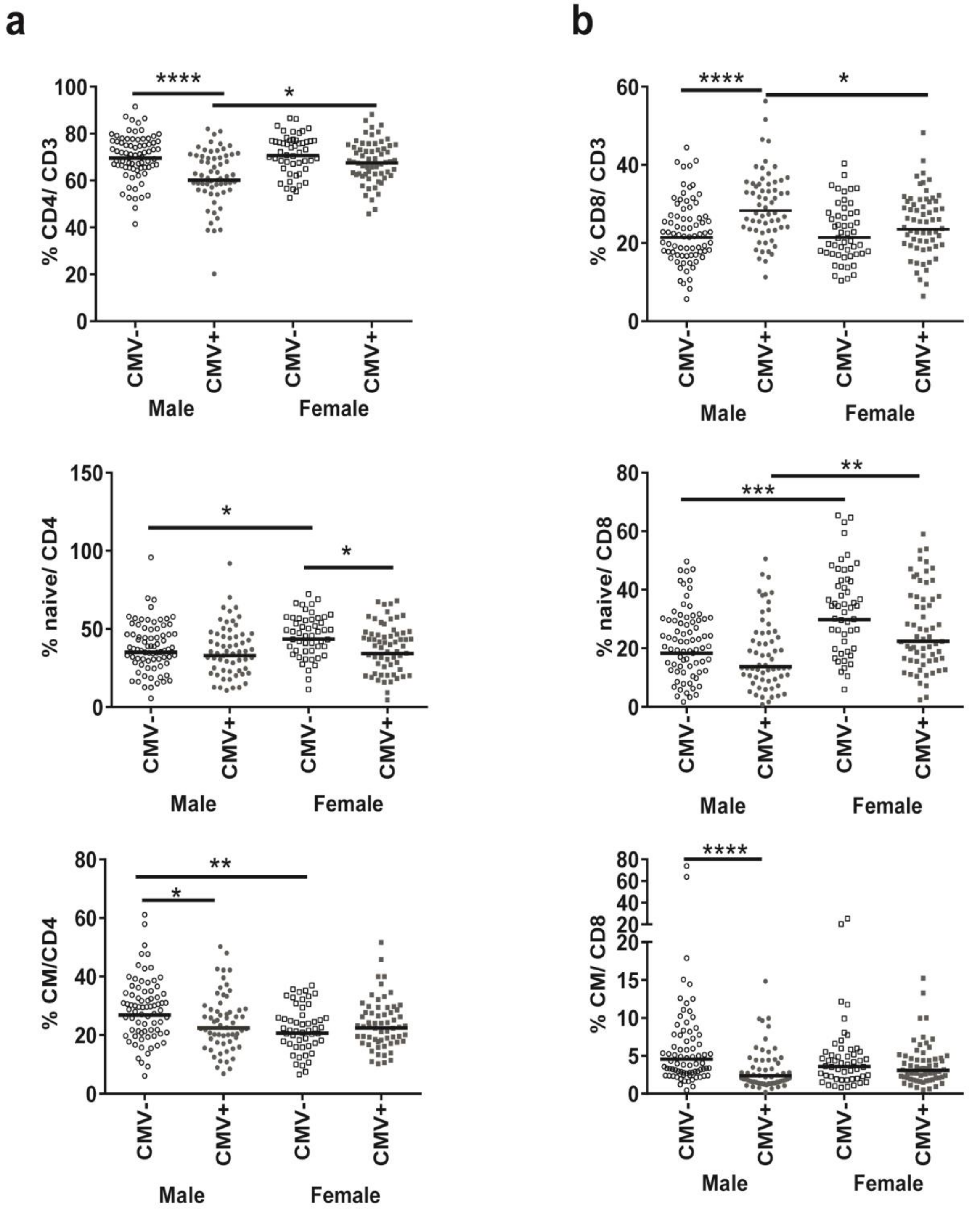

Supplementary figure 3. The relative numbers of total CD4, CD8, naive and CM T-cells
a) The relative proportion of total CD4, naïve CD4, and CM CD4 T-cells within the total CD3+ T-cell population.
b) The relative proportion of total CD8, naïve CD8, and CM CD8 T-cells within the total CD3+ T-cell population.
The Geometric mean is indicated in the graphs, and the Kruskal-Wallis test was used for statistical analysis. * $p<0.05$, ** $p<0.01$, *** $p<0.001$, **** $p<0.0001$ after Bonferroni correction (n=250).

Supplementary Table 1. General health status of the study cohort (n = 255), separated by gender and CMV status.

medication used more than 3 months ago. a. CMV- male vs CMV- female, b. CMV+ male vs CMV+ female, c. CMV- female vs CMV+ female. *p<0.05, **p<0.01, ***p<0.001, ****p<0.0001. Bold numbers indicate significant differences within one measurement. After correction for multiple testing only values with p< 0.0014 are considered significant. Significant differences are underlined. The Chi Square test was used to determine significant differences in frequencies between the groups. Biochemical parameters were compared with the Kruskal-Wallis test (adjusted for multiple comparisons).

	CMV+ male	CMV- male	CMV+ female	CMV- female
**Diseases in last year (number)**				
Diabetes type II	4	6	2	**0** *a
High blood pressure	5	9	**14** *b	7
Vascular diseases	1	4	2	2
Lung diseases	2	4	3	0
Rheumatic diseases	2	2	0	0
Gastro-intestinal diseases	1	3	1	0
Liver diseases	0	0	0	0
Other diseases	3	4	5	2
No serious diseases	49	56	**38** *b	39
**Medication last 6 months (number)**				
Medication for infections #	4	5	8	3
Cholesterol lowering medication	6	10	12	4
Diabetic medication	2	5	1	0
Blood pressure lowering medication	7	18	15	8
Immunosuppressive medication #	1	1	2	0
No medication	47	54	**33** **b/ **C	39
**Infections (number)**				
Influenza <4 weeks	2	3	3	2
Cold <4 weeks	14	13	7	6
No infection < 4 weeks	45	63	53	41
**Smoking (number)**				
Cigarette smoking	9	10	7	7
Cigars, pipe smoking	2	4	2	1
None smoking	49	63	53	42
**Physical activity (numbers)**				
Weekly or more	42	51	42	36
Less than weekly	7	10	10	4
No activity	11	17	10	9
**Biochemical parameters**				
CRP (mg/L)	1.15	1.0	1.55	1.43
Geomean [95% CI]	[0.9 – 1.5]	[0.8 – 1.2]	[1.2 – 2.0]	[1.0 – 1.9]
DHEAs (μmol/L)	3.9	3.7	**2.4** ****b	**2.1** ***a
Geomean [95% CI]	[3.4 – 4.5]	[3.2 – 4.3]	**[2.0 – 2.8]**	**[1.8 – 2.4]**
Rheumatoid Factor (IU/ml)	4.0	3.6	3.4	2.9 *a
Geomean [95% CI]	[3.3 – 4.8]	[3.1 – 4.2]	[2.7 – 4.1]	[2.7 – 4.1]
Reactive Oxygen Metabolites (IU/L)	333.4	333.8	**390.1** ****b	**398.1** ****a
Geomean [95% CI]	[317.3 – 350.4]	[321.3 – 346.9]	**[373.1 – 407.9]**	**[382.3 – 414.5]**

Supplementary Table 2. Linear regression analysis for the absolute numbers of immune cells

Linear (Enter) regression method is used. Variables: CMV status (0= seronegative, 1= seropositive), Gender (0=female, 1= male), Gender* CMV status, and Age (years). All data are log-transformed. *p<0.05. Bold numbers indicate significant differences within one subset. After a multiple testing correction only values with p< 0.0014 are considered significant. Significant differences after correction are underlined.

Cell subset	Predicting variable	p-value	β coefficient	R^2 model
T cell	CMV status	0.928	-0.008	0.108
	**Gender**	**0.016***	**-0.209**	
	Gender*CMV	0.124	-0.162	
	Age	0.422	0.049	
CD4 TemRO early	CMV status	0.873	-0.015	0.030
	Gender	0.666	0.039	
	Gender*CMV	0.099	-0.181	
	Age	0.905	0.008	
CD4 TemRO int	CMV status	0.325	0.094	0.010
	Gender	0.707	0.034	
	Gender*CMV	0.189	-0.145	
	Age	0.929	-0.006	
CD4 TemRA early	CMV status	0.474	-0.066	0.059
	**Gender**	**0.021***	**-0.205**	
	Gender*CMV	0.692	-0.043	
	Age	0.478	0.044	
CD4 TemRA int	CMV status	0.967	-0.004	0.035
	**Gender**	**0.015***	**-0.219**	
	Gender*CMV	0.244	0.127	
	Age	0.989	-0.001	
CD8 TemRO early	CMV status	0.876	-0.015	0.030
	Gender	0.661	0.039	
	Gender*CMV	0.098	-0.181	
	Age	0.905	0.008	
CD8 TemRO int	CMV status	0.326	0.094	0.010
	Gender	0.707	0.034	
	Gender*CMV	0.189	-0.145	
	Age	0.929	-0.006	
CD8 TemRA early	CMV status	0.558	-0.055	0.030
	Gender	0.356	0.083	
	Gender*CMV	0.170	-0.150	
	Age	0.915	0.007	
CD8 TemRA int	CMV status	0.967	-0.004	0.035
	**Gender**	**0.015***	**-0.219**	
	Gender*CMV	0.244	0.127	
	Age	0.989	-0.001	
CD8/CD4 ratio	CMV status	0.181	-0.122	0.090
	Gender	0.936	0.007	
	Gender*CMV	0.061	-0.199	
	Age	0.101	0.100	
Treg	CMV status	0.519	-0.059	0.076

	Gender	0.143	-0.129	
	Gender*CMV	0.141	-0.157	
	Age	0.486	0.043	
CD4+CD45RA+ CD25dim	CMV status	0.301	-0.096	0.070
	Gender	0.060	-0.166	
	Gender*CMV	0.472	-0.077	
	Age	0.120	0.097	
naive Treg	CMV status	0.245	-0.108	0.060
	**Gender**	**0.006***	**-0.245**	
	Gender*CMV	0.852	0.020	
	Age	0.701	-0.024	
memory Treg	CMV status	0.084	-0.162	0.049
	Gender	0.913	-0.010	
	Gender*CMV	0.490	-0.075	
	Age	0.349	0.059	
B cell	CMV status	0.778	-0.027	0.032
	Gender	0.256	-0.102	
	Gender*CMV	0.419	-0.088	
	Age	0.411	0.052	
transitional	**CMV status**	**0.040***	**-0.194**	0.038
	**Gender**	**0.024***	**-0.203**	
	Gender*CMV	0.366	0.098	
	Age	0.932	-0.005	
naive mature	CMV status	0.355	-0.088	0.021
	Gender	0.203	-0.115	
	Gender*CMV	0.985	-0.002	
	Age	0.403	0.053	
natural effector	CMV status	0.116	0.149	0.020
	Gender	0.801	0.023	
	Gender*CMV	0.136	-0.164	
	Age	0.373	-0.057	
CD27- memory	CMV status	0.799	-0.024	0.054
	Gender	0.088	-0.152	
	Gender*CMV	0.508	-0.072	
	Age	0.057	0.119	
CD27+ memory	CMV status	0.126	0.143	0.049
	Gender	0.370	-0.080	
	Gender*CMV	0.120	-0.169	
	Age	0.238	0.074	
